# Absence of Arabidopsis Polyamine Oxidase 5 Influences the Cytokinin-Induced Shoot Meristem Formation from Lateral Root Primordia

**DOI:** 10.3390/plants12030454

**Published:** 2023-01-18

**Authors:** Nikolett Kaszler, Péter Benkő, Árpád Molnár, Abigél Zámbori, Attila Fehér, Katalin Gémes

**Affiliations:** 1 Institute of Plant Biology, Biological Research Centre, Eötvös Loránd Research Network, 62. Temesvári krt., H-6726 Szeged, Hungary; 2 Doctoral School of Biology, University of Szeged, 52. Közép fasor, H-6726 Szeged, Hungary; 3 Department of Plant Biology, University of Szeged, 52. Közép fasor, H-6726 Szeged, Hungary

**Keywords:** *Arabidopsis thaliana*, direct shoot regeneration, nitric oxide, polyamine oxidase, haemoglobins, cytokinin sensitivity

## Abstract

Lateral root primordia (LRPs) of Arabidopsis can be directly converted to shoot meristems (SMs) by the application of exogenous cytokinin. Here, we report that Arabidopsis POLYAMINE OXIDASE 5 (AtPAO5) contributes to this process, since the rate of SM formation from LRPs was significantly lower in the *pao5-2* knockout mutant. Furthermore, the presented experiments showed that AtPAO5 influences SM formation via controlling the thermospermine (T-Spm) level. Gene expression analyses supported the view that the *pao5-2* mutation as well as exogenous T-Spm downregulate the expression of the class 3 haemoglobin coding genes *AtGLB1* and *AtGLB2*. AtGLB1 and 2 have been reported to augment cytokinin sensitivity, indirectly inhibiting the expression of type-A ARABIDOPSIS RESPONSE REGULATORs (ARRs). In agreement, the same ARR-coding genes were found to be upregulated in the *pao5-2* mutant. Although GLB proteins might also control cytokinin-induced nitric oxide (NO) accumulation, we could not find experimental evidence for it. Rather, the negative effect of NO-donor treatment on AtPAO5 gene expression and SM formation was seen. Nevertheless, a hypothetical pathway is set up explaining how AtPAO5 may affect direct shoot meristem formation, controlling cytokinin sensitivity through T-Spm and GLBs.

## 1. Introduction

Plant organogenesis is one of the tissue culture pathways for plant propagation. During this process, plants can be regenerated from various explants via de novo shoot meristem formation. The success of in vitro regeneration depends on several factors, such as the suitable genotype, explant, proper environmental condition, and hormone combination. Most of these regeneration systems are indirect and involve auxin-induced callus formation followed by cytokinin-induced shoot meristem regeneration [1,2]. However, lateral root primordia (LRPs) of Arabidopsis can be converted to shoot meristems (SMs) without callus formation by exogenous cytokinin application [3,4,5,6,7]. The most efficient direct shoot organogenesis system was set up by Rosspopoff et al. (2017). In this system, auxin-induced LRPs of excised Arabidopsis roots could be converted to SMs in the presence of cytokinin. The same system also worked with roots of whole seedlings avoiding the wounding step [7]. In these systems, the conversion of LRPs to SMs starts with a mitotic pause (at around 24 h after cytokinin induction), during which cell divisions are transiently arrested in the competent roots. Following this mitotic pause, LRPs go through a conversion phase (at around 48 h of cytokinin induction) and from the converting organs early (at around 72 h) and late (at around 96 h) shoot promeristems develop [5,7].

The process of de novo shoot regeneration involves the perception of cytokinin, its signalling, and the transcriptional activation of the *WUSCHEL* (*WUS*) gene [8]. *WUS* plays a major role in the formation of shoot stem-cell niches, which subsequently generate signals that maintain the equilibrium between self-renewal and the production of daughter cells that can differentiate into various tissues [9]. Therefore, the transcriptional activation of *WUS* expression is a critical molecular event for CK-induced shoot organogenesis. In Arabidopsis plants, cytokinin signalling is mediated by histidine kinase receptors, phosphorelay shuttle proteins, and type-B response regulators. Type-A response regulators provide negative feedback on the multistep-phosphorelay signalling [10]. The role of cytokinin signalling in de novo shoot regeneration has recently been discussed in detail by Hnatuszko-Konka et al. [11]. Cytokinin responses during shoot regeneration have been shown to be modulated by various pathways, such as the polyamine metabolism [7], haemoglobin expression [12], and NO levels [13].

PA synthesis and especially the level of spermidine (Spd) was shown to be increased during the LRP-to-SM conversion on cytokinin-induced Arabidopsis roots and the application of exogenous Spd improved the efficiency of the conversion [7]. Polyamines (PAs) were showed to interact with auxin and cytokinin during other developmental processes [14,15]. Beside their synthesis, their catabolism also has a crucial role in plant growth and development [16]. Polyamine catabolism is mediated mainly by two types of amine oxidases (AOs). One of them is diamine oxidases (DAOs), which oxidize mainly putrescine (Put), but also spermidine (Spd), and spermine (Spm) (with much lower efficiency), yielding hydrogen peroxide (H_2_O_2_) and aminoaldehydes. The others are polyamine oxidases (PAOs). Certain PAOs terminally oxidize Spd and Spm, but not Put, also yielding H_2_O_2_ and aminoaldehydes [17,18]. Other PAO enzymes can also catalyse the backconversion of tetraamine to triamine and/or triamine to diamine, also producing H_2_O_2_ beside altering PA levels/ratios [17,18]. The Arabidopsis genome has five genes (*AtPAO1*–AtPAO5) coding for PAO enzymes among which AtPAO5 preferentially catalyses the backconversion of T-Spm to Spd [19]. Moreover, AtPAO5 has dehydrogenase rather than oxidase activity; therefore, it was hypothesized that AtPAO5 contributes to the regulation of plant growth, xylem differentiation, and direct shoot meristem formation via controlling the T-Spm homeostasis [7,19,20]. Notably, AtPAO5 was hypothesized to affect these processes altering the auxin–cytokinin cross talk [7,19].

Wang et al. showed that class 3 haemoglobins (AtGLBs) are involved in shoot organogenesis [12]. Overproduction of AtGLB1 (*35S::GLB1*) and AtGLB2 (*35S::GLB2*) improved the efficiency of indirect shoot meristem formation, while the repression of AtGLB2 inhibited it. Manipulating the AtGLB1 or 2 levels altered the transcript abundance of genes participating in cytokinin perception and signalling. This increased the cytokinin sensitivity of the Arabidopsis roots. Haemoglobins can scavenge nitric oxide (NO) that may mediate their effect on cytokinin signalling as on other plant signalling and developmental processes [21].

Nitric oxide (NO) is a gaseous redox signalling molecule with diverse regulatory roles in plants [22,23]. In plants, NO can be synthesized enzymatically in reductive or oxidative pathways, or non-enzymatically [24,25]. Among the reductive enzymatic pathways, the most relevant is the reduction of nitrate to nitrite and nitrite to NO by nitrate reductase (NR) [26]. Therefore, the NR-deficient Arabidopsis *nia1nia2* mutant [27] was often used to investigate the role of NO in plant development and adaptation (e.g., [28,29]). Alternatively, treatment of plants with NO-donor molecules, such as S-nitrosoglutathione (GSNO), was used to study the effect of NO on various processes, including regeneration and morphogenesis. In this way, NO was shown, for example, to enhance the indirect shoot regeneration in vanilla [30] and snapdragon [31], and the root formation in cucumber and tomato [32,33] in a dose-dependent manner.

While NO regulates adventitious root formation [32] and lateral root development through the mediation of auxin action [33], it is also involved in cytokinin responses. In Amaranthus seedlings, the cytokinin effect on betacyanin accumulation was mimicked by NO donors [34]. However, exogenous application of NO did not alter the cytokinin-responsive expression of *ARR5* in Arabidopsis, which suggests the indirect role of NO in triggering cytokinin responses [35]. Moreover, cytokinin was shown to induce NO generation through the activation of the NR NIA1 [36]. Cytokinin participates in the regulation of the cell cycle inducing the expression of the gene coding for CYCLIN D3;1 (*CYCD3;1*), which was shown to be dependent on NO accumulation [13]. These observations support the view that NO acts downstream of cytokinin in the induction of cell proliferation. In agreement, it was reported that the roots of the *nos1*/*noa1* Arabidopsis mutant with reduced NO level showed reduced callus formation and indirect shoot regeneration that could be complemented by *CYCD3;1* overexpression [13].

PAs, especially Spd and Spm, can promote NO production [37,38,39,40]. Moreover, L-arginine, a precursor of polyamine biosynthesis, can serve as a source of NO [41]. NO formation was also linked to PA catabolism but in a yet unknown way [41].

Therefore, there are several experimental data suggesting that PAs, AtPAO5, haemoglobins, and NO might be interrelated in affecting cytokinin responses. In this study, our aim was to investigate this possibility, investigating a strongly cytokinin-dependent process, i.e., the direct shoot meristem formation from Arabidopsis LRPs.

## 2. Materials and Methods

### 2.1. Plant Material, Growth Condition, and Treatments

Seedlings of *Arabidopsis thaliana* wild type of the ecotype Columbia (Col-0) and *pao5-2* T-DNA insertion mutant obtained from SALK collection (SALK_053110) were used for the experiments. All plants were grown in a climate-controlled cabinet using 8/16 (light/dark) photoperiod, a constant temperature of 21 °C, with an irradiance of 50 µmol m^−2^ s^−1^ provided by white fluorescent tubes (Sylvania Luxline Plus; Feilo Sylvania Europe Limited, London, UK). To induce direct organogenesis, the method of Rosspopoff et al. (2017) was used with minor modifications (7). Surface-sterilized seeds (70% ethanol for 60 s followed by immersion for 10 min in 4% commercial sodium hypochlorite solution having 4.5% active chlorine) were germinated and the seedlings were grown for 6 days on solid medium containing full-strength MS (Murashige and Skoog Medium including B5 vitamins, Duchefa Biochemie, Haarlem, The Netherlands), 1% sucrose (Molar Chemicals, Halásztelek, Hungary), 0.6% agarose (Electran DNA pure grade for electrophoresis: VWR International LLC, Radnor, PA, USA), 0.5 g/L 2-(N-morpholino)ethanesulfonic acid (MES) (Duchefa Biochemie). For the priming of lateral root primordia (LR) initiation, 3.3 µM naphthaleneacetic acid (NAA) (Duchefa Biochemie) was applied for 43 h. To synchronize LR initiation, 1.25 µM 2,3,5-triiodobenzoic acid (TIBA) (Fluka, Chemie GmbH, Buchs, Switzerland) was added to the germination medium before the NAA treatment. To induce the conversion of lateral root primordia (LRPs) into functional shoot meristems (SMs), seedlings were transferred onto and cultured on a medium (SM medium) containing full-strength MS salts (Duchefa Biochemie), 2% D(+)-glucose (Molar Chemicals), 0.6% agarose (Electran DNA pure grade for electrophoresis), and 8.16 µM isopentenyladenine (IPA) (Sigma-Aldrich, St. Louis, MO, USA). Stock solutions of T-Spm, H_2_O_2_, and GSNO were prepared in Milli-Q^®^ H_2_O, filter sterilized with Millex^®^ GV syringe filter (0.22 µm; Merck Millipore, Burlington, MA, USA), and added to the SM medium at 100 µM (H_2_O_2_ and T-Spm) or 10 µM (GSNO) final concentration. Samples were collected from roots of the seedlings after 24, 48, 72, and 96 h cytokinin induction. As absolute control, roots of 6-day-old, nontreated seedlings were used.

### 2.2. Light Microscopy

To determine the conversion efficiency of LRPs to SMs, Olympus SZX12 stereo dissection microscope (Olympus Corporation, Sindzsuku, Tokyo, Japan) was used with a white LED light source (Photonic Optics, Vienna, Austria). Photos were captured using an Olympus Camedia C7070 digital camera (Olympus Corporation) and the DScaler software (version 4.1.15).

### 2.3. RT-PCR and Quantitative Real-Time RT-PCR Analyses

For total RNA extraction, the Quick-RNA Miniprep Kit (Zymo Research, Irvine, CA, USA) was used including the reagents to remove any contaminating genomic DNA. NanoDrop™ 2000/2000c spectrophotometer (Thermo Fisher Scientific, Waltham, MA, USA) was used to evaluate the quality and quantity of total isolated RNA (1.8 ≤ A260/280 ≤ 2.0). In total, 350 ng of total RNA was reverse-transcribed for 60 min at 42 °C and for 10 min at 75 °C in a 20 µL reaction volume using RevertAid First Strand cDNA Synthesis Kit (Thermo Fisher Scientific) according to the manufacturer’s instructions. cDNA products were diluted 1:10 in AccuGENE^®^ water (Lonza, Verviers, Belgium). Primers were designed using the Primer 3 software [42] and synthesized by Biocenter Ltd. (Szeged, Hungary). Primer sequences are shown in Supplementary Table S1. Primer sequences were analysed using OligoAnalyzerTM Tool (Integrated DNA Technologies, Inc., Coralville, IA, USA) and National Centre for Biotechnology Information (NCBI) programs (Bethesda (MD): National Library of Medicine (US), National Centre for Biotechnology, 1982). Relative mRNA levels were determined by real-time quantitative PCR (RT-qPCR). As reference genes, *UBIQUITIN 1* (At3G52590) and *PP2A3* (At1G13320) were used. These genes were selected using the Arabidopsis Regeneration eFP browser at the Bio-Analytic Resource for Plant Biology (bar.u-toronto.ca; [43]) allowing the in silico analysis of transcriptomic data sets of root-to-shoot regeneration experiments. According to these data, the At3G52590 and At1G13320.1 genes have constitutive expression during the process. The RT-qPCR reactions were carried out by the CFX384 Touch Real-Time PCR Detection System (BioRad Laboratories Inc., Hercules, CA, USA). The PCR mixture contained (in a total volume of 7 µL) 1 µL cDNA, 0.21 µL forward primer, 0.21 µL reverse primer, 3.5 µL Maxima SYBR Green/ROX qPCR Master Mix (2×) (Thermo Fisher Scientific). Reaction mixtures were aliquoted to Hard-Shell^®^ 384-well plates (thin-wall, skirted, clear/white; Bio-Rad, Cat. no: HSP3805). For amplification, a standard two-step thermal cycling profile was used (10 s at 95 °C and 1 min at 60 °C) during 40 cycles, after a 15 min preheating step at 95 °C. Finally, a dissociation stage was added with 95 °C for 15 s, 60 °C for 15 s, and 95 °C for 15 s. Data analysis was performed using Bio-Rad CFX Maestro (Bio-Rad) software and Microsoft Excel 2010. The relative mRNA levels were calculated using the (2)^−∆∆Ct^ method. mRNA level of the initial root tissue was used as reference (relative mRNA level: 1). All tested amplification efficiencies were in a narrow range and were not used in the data normalization. Data were averaged from three independent biological experiments with three technical replicates for each gene/sample combination.

### 2.4. In Situ Detection of Reactive Oxygen Species (ROS) and Nitric Oxide

For in situ detection of ROS, 2,7-dichlorodihydrofluorescein diacetate (H2DC-FDA, Sigma-Aldrich) was used. Seedling roots were incubated in 10 µM H2DC-FDA solubilized in 2-Nmorpholine-ethansulphonic acid/potassium chloride (MES/KCl) pH 6.5 for 15 min at room temperature in darkness. After staining, seedling roots were washed once with a dye-free buffer and the fluorescence of the oxidized product of H2DC-FDA, dichlorofluorescein (DCF), was visualized by a fluorescent microscope.

For in situ detection of NO, 4-amino-5-methylamino-2′, 7′-difluorofluorescein diacetate (DAF-FM DA, Abcam, Cambridge, UK) was applied. After the incubation of the seedling roots in 10 μM DAF-FM DA for 30 min in darkness at room temperature, the excess fluorophore was washed out with Tris-HCl buffer (10 mM, pH 7.4) [40,44].

To detect fluorescence intensity, a Zeiss Axiowert 200 M-type fluorescent microscope (Carl Zeiss, Germany) equipped with a high-resolution digital camera (Axiocam HR) was used. The fluorescence intensity was estimated by the Axiovision Rel. 4.8 software using a filter set 10 (excitation at 450−490 nm and emission detection at 515−565 nm). The same camera settings were used for each digital image. Means of pixel intensities were calculated within the region of interest of 25 µm diameter circles at mitotic pause (after 24 h cytokinin induction), 30 µm diameter circles at the converting organ (after 48 h cytokinin induction), 40 µm diameter circles at early and 60 µm diameter circles at the late shoot promeristem (72 and 96 h after cytokinin induction) to cover the whole middle region of the converting organ/promeristem (Supplementary Figure S1). The relative fluorescence intensities of at least 20 converting LRPs/shoot promeristems in each of the three replicates were measured. The average fluorescence intensities of converting organs/promeristems with the same region of interest in the control and mutant/treated roots at the same stage were pairwise compared and analysed statistically. The average of the control untreated roots was selected as the reference (relative pixel intensity unit 1). Absolute data are provided in Supplementary Tables S2 and S3.

### 2.5. Statistical Analysis

Statistical analysis was performed using the SIGMAPLOT12.0 statistical software. Quantitative data are presented as the mean ± SE and the significance of difference between sets of data was determined by one-way analysis of variance (ANOVA) following Duncan’s multiple range tests; *p*-values of less than 0.05 were considered significant. For pairwise comparisons, Student’s *T*-test was used (* *p* ≤ 0.05, ** *p* ≤ 0.01, *** *p* ≤ 0.001)

## 3. Results

### 3.1. AtPAO5 Influences the Efficiency of Direct Shoot Meristem Formation via Controlling T-Spm Levels

**Figure 1:**
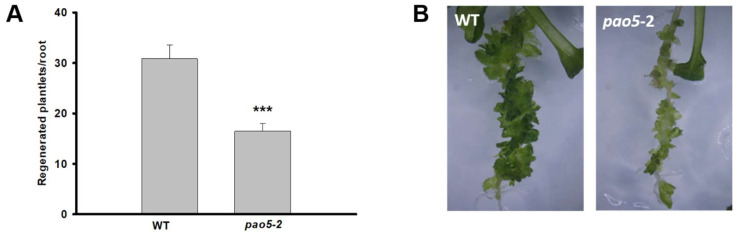
*pao5-2* T-DNA insertion mutant plants show a decrease in the efficiency of the conversion of lateral root primordia into shoots. (**A**) Shoot regeneration rate of seedling roots of wild type (WT) and *pao5-2* mutants after 10 days of cytokinin induction. Data are means ± SE of three biological replicates with twenty technical replicates each. For significance analysis Student’s *T*-test was used. (*** *p* ≤ 0.001). (**B**) Representative images of shoot-regenerating seedling roots.

**Figure 2:**
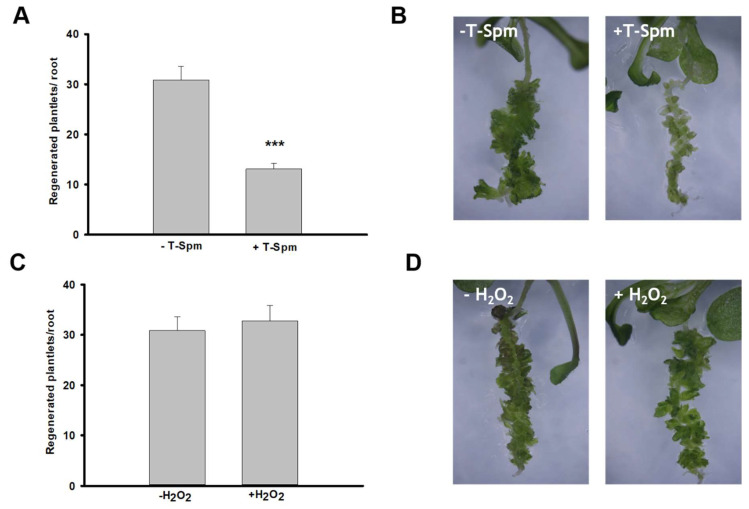
Conversion of lateral root primordia to shoot meristem is affected by exogenous T-Spm but not by H_2_O_2_. (**A**) Effect of exogenously applied T-Spm (+T-Spm) on the shoot regeneration efficiency of untreated WT seedling roots (−T-Spm) after 10 days of cytokinin induction. (**B**) Representative images of shoot-regenerating roots used for the calculation shown in A. (**C**) Effect of exogenously applied 100 µM H_2_O_2_ (+H_2_O_2_) on the shoot regeneration efficiency of untreated WT seedling roots (−H_2_O_2_) under the same conditions as in A. (**D**) Representative images of shoot regenerating roots used for the calculation shown in C. In A and C, data are means ± SE of three biological replicates with twenty technical replicates each. For significance analysis Student’s *T*-test was used. (*** *p* ≤ 0.001).

The positive effect of AtPAO5 overexpression on the conversion of LRPs to SMs has been documented in our earlier study [7]. However, the mechanism by which AtPAO5 might regulate the process was not revealed. AtPAO5 has high affinity for T-Spm, and the *pao5-2* mutant plants have 2-fold higher T-Spm than the wild type (WT) [20]. Furthermore, T-Spm was shown to negatively affect plant growth and development [19,20]. Therefore, it was hypothesized that AtPAO5 influences direct shoot meristem formation via controlling the T-Spm level [7]. To verify this hypothesis, we investigated the LRP-to-SM conversion potential of *pao5-2* mutants. In agreement with the *35S::AtPAO5* overexpression phenotype, we found that the regeneration rate of the *pao5-2* mutant was significantly lower than that of the WT plants (Figure 1). Furthermore, not exogenously applied H_2_O_2_ but T-Spm decreased the efficiency of direct shoot meristem formation in comparison to the untreated controls (Figure 2). In agreement, *pao5-2* and WT plants did not show difference in ROS accumulation during the conversion of LRPs to SMs (Supplementary Figure S2). These results are in line with our hypothesis that AtPAO5 influences the shoot regeneration potential of Arabidopsis roots controlling the T-Spm homeostasis.

### 3.2. The pao5-2 Mutation and Exogenous T-Spm Might Decrease Cytokinin Sensitivity through the Downregulation of AtGLB1/2-Coding Genes

**Figure 3:**
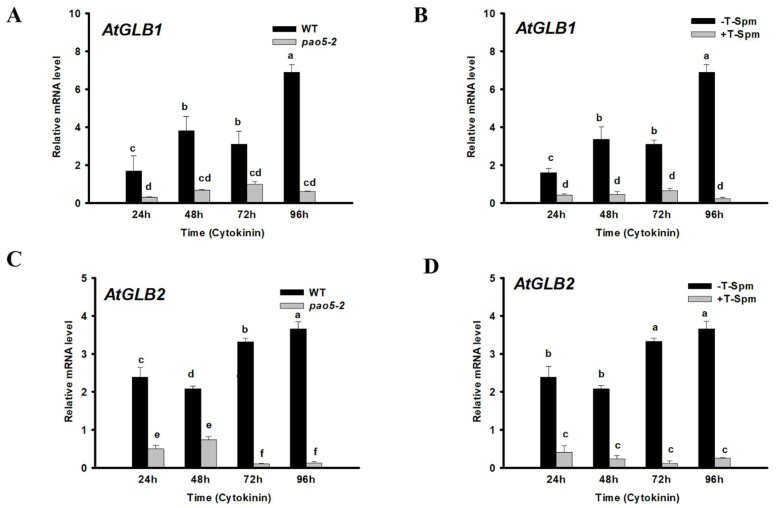
Expression of genes coding the class 3 haemoglobins AtGLB1 (**A**,**B**) and 2 (**C**,**D**) in wild-type (WT), *pao5-2* mutant, and T-Spm-treated/untreated wild-type (+−T-Spm) Arabidopsis roots induced to regenerate shoots. Samples taken at 24, 48, 72, and 96 h after cytokinin induction represent the mitotic pause (24 h), organ conversion (48 h), early (72 h) and late (96 h) shoot promeristem phases. The mRNA levels of the *UBIQUITIN 1* (At3G52590) and *PP2A3* (At1G13320.1) genes were used for gene expression normalization. The mRNA level of the untreated root tissue was used as reference (relative mRNA level: 1). Data were averaged from three independent biological experiments with three technical replicates each. Standard errors are shown on the columns. The significance of difference between sets of data was determined by one-way analysis of variance (ANOVA) following Duncan’s multiple range tests; a *p*-value of less than 0.05 was considered significant. Different letters indicate significant differences.

**Figure 4:**
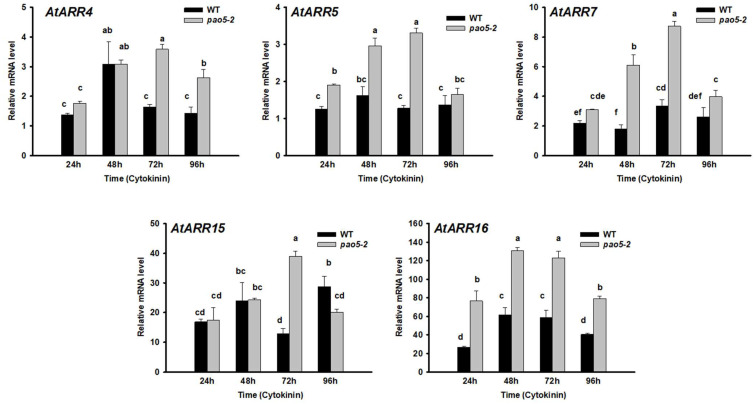
Relative mRNA levels of genes coding for type-A Arabidopsis response regulators (AtARRs) during shoot organogenesis in wild type (WT) and *pao5-2* mutants after 24, 48, 72, and 96 h cytokinin induction. The mRNA levels of the *UBIQUITIN 1* (At3G52590) and *PP2A3* (At1G13320.1) genes were used for gene expression normalization. The mRNA level of the untreated root tissue was used as reference (relative mRNA level: 1). Data were averaged from three independent biological experiments with three technical replicates each. Standard errors are shown on the columns. The significance of difference between sets of data was determined by one-way analysis of variance (ANOVA) following Duncan’s multiple range tests; a *p*-value of less than 0.05 was considered significant. Different letters indicate significant differences.

AtPAO5 was reported to influence cytokinin responses in a T-Spm-dependent manner during xylem differentiation in a yet unknown way [19]. Since the haemoglobins AtGLB1 and AtGLB2 were reported to affect cytokinin sensitivity during indirect shoot regeneration [12], we investigated their expression under the direct SM regeneration process in WT and *pao5-2* mutant roots. Samples were collected at timepoints marking the four main stages of the conversion of LRPs to SMs: mitotic pause (at 24 h), converting organ phase (at 48 h), and the development of early (at 72 h), and late shoot promeristem (at 96 h) [5,7]. It was found that both genes had increased expression especially during the promeristem stage (72–96 h after cytokinin induction), but the increase in the relative expression of *AtGLB1* started earlier and was more pronounced than that of *AtGLB2* (Figure 3). The loss of AtPAO5 function known to increase the endogenous T-Spm level as well as exogenous T-Spm treatment decreased the relative mRNA level of both *AtGLB1* and *AtGLB2* at all timepoints (Figure 3). Overexpression of *AtGLB1* and *AtGLB2* was reported to increase cytokinin sensitivity through the repression of A-type ARRs, which are feed-back repressors of the cytokinin signalling pathway [12]. In accordance, the relative transcript level of A-ARRs (*AtARR5*, *AtARR7*, *AtARR16*) was enhanced in the *pao5-2* mutant roots after 24 h cytokinin induction and remained high until the formation of late promeristems (96 h cytokinin induction) in comparison to the WT roots (Figure 4). The mRNA levels of *AtARR4* and *AtARR15* were also higher in the *pao5-2* mutant than in the WT but only at the formation of shoot promeristems (72 h CK induction) (Figure 4). Altogether, this shows that AtPAO5 might control cytokinin sensitivity, at least partly, via T-Spm-dependent *AtGLB1/2* expression.

### 3.3. The Effect of NO on AtPAO5-Dependent LRM-to-SM Conversion Efficiency

Since AtGLBs are potent in planta NO scavengers [21], and the effect of NO on cytokinin signalling can be synergistic or antagonistic [45], we decided to investigate whether NO is also involved in the cytokinin-induced direct shoot regeneration process in an AtPAO5-dependent manner.

**Figure 5:**
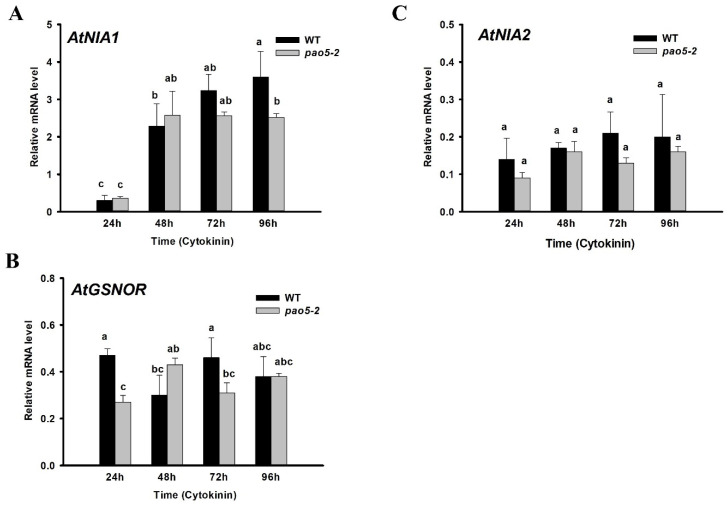
Expression of genes coding for enzymes implicated in nitric oxide homeostasis during the direct formation of shoot meristem from lateral root primordia. The relative transcript levels of *AtNIA1* (**A**), *AtNIA2* (**B**), and *AtGSNOR* (**C**) were investigated at the mitotic pause (24 h), organ conversion (48 h), early (72 h) and late (96 h) shoot promeristem phases. The mRNA levels of the *UBIQUITIN 1* (At3G52590) and *PP2A3* (At1G13320.1) genes were used for gene expression normalization. The mRNA level of the untreated root tissue was used as reference (relative mRNA level: 1). Data were averaged from three independent biological experiments with three technical replicates each. Standard errors are shown on the columns. The significance of difference between sets of data was determined by one-way analysis of variance (ANOVA) following Duncan’s multiple range tests; a *p*-value of less than 0.05 was considered significant. Different letters indicate significant differences.

**Figure 6:**
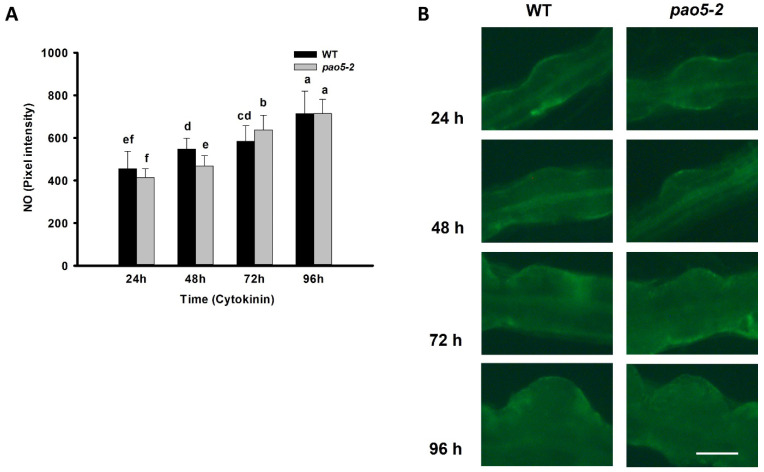
Detection of NO-dependent fluorescence of DAF-FM DA-stained wild-type (WT) and *pao5-2* mutant roots after 24, 48, 72, and 96 h cytokinin induction representing the mitotic pause (24 h), organ conversion (48 h), early (72 h) and late (96 h) promeristem phases of shoot meristem regeneration. (**A**) Pixel intensity of the LRPs stained with the fluorescent dye. Data are means ± SE of three biological replicates with twenty technical replicates each. The significance of the difference between samples was analysed by one-way analysis of variance (ANOVA) following Duncan’s multiple range tests; *p*-values of less than 0.05 were considered significant. Different letters indicate significant differences. (**B**) Visualization of NO-dependent DAF-FM DA fluorescence during the direct conversion of lateral root primordia to shoot meristem using fluorescence microscopy. Scale bar = 100 μm.

The expression level of various genes coding for enzymes involved in NO metabolism was investigated in Arabidopsis roots after 24, 48, 72, and 96 h of cytokinin induction. The relative transcript level of *AtNIA1* significantly increased in all stages of shoot meristem formation of WT as well as *pao5-2* mutant plants, although the increase was lower at the promeristem stage in the mutant (Figure 5). In contrast, the relative mRNA level of *AtNIA2* did not change during the conversion of LRPs to SMs, nor of the WT or *pao5-2* mutants (Figure 5B). The relative expression of *AtGSNOR* coding for S-NITROSOGLUTATHIONE REDUCTASE being implicated in the maintenance of NO homeostasis did not change during the formation of shoot promeristems, neither in the WT nor in the mutant roots (Figure 5C). In agreement with the limited or no difference in the expression of genes involved, we could detect only a limited and transient difference at 48–72 h in the in situ NO levels of WT and *pao5-2* mutant roots during the formation of SMs (Figure 6). Nevertheless, since the expression of *AtNIA1* increases app. three-fold during the regeneration process in both the WT and the mutant, the potential role of NO in cytokinin signalling cannot be excluded.

**Figure 7:**
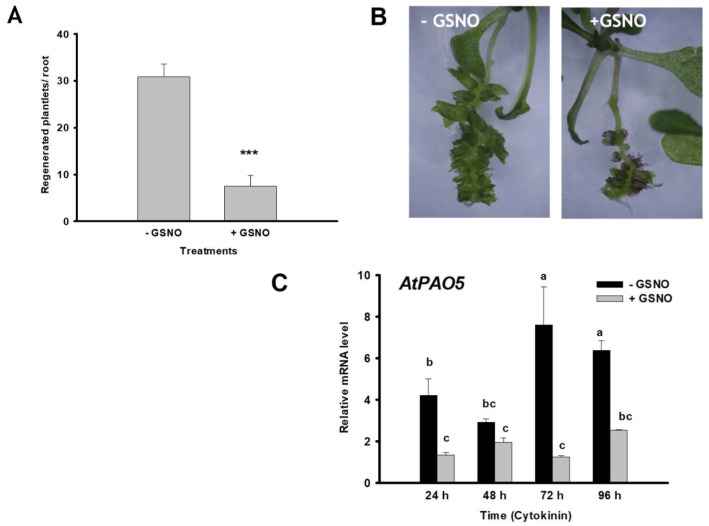
Exogenous nitric oxide (GSNO) decreases both the efficiency of the direct formation of shoot meristem from lateral root primordia in Arabidopsis seedlings as well as the relative mRNA level of the AtPAO5 gene. (**A**) Shoot regeneration in GSNO-treated and non-treated Arabidopsis seedling roots after 10 days cytokinin induction. Data are means ± SE of three biological replicates with twenty technical replicates each. For significance analysis Student’s T-test was used. (*** *p* ≤ 0.001). (**B**) Representative images of regenerating seedling roots. (**C**) Relative expression level of the AtPAO5 gene during direct organogenesis of GSNO-treated and non-treated seedling roots of Arabidopsis at the mitotic pause (24 h), organ conversion (48 h), early (72 h) and late (96 h) shoot promeristem phases. The mRNA levels of the *UBIQUITIN 1* (At3G52590) and *PP2A3* (At1G13320.1) genes were used for gene expression normalization. The mRNA level of the untreated root tissue was used as reference (relative mRNA level: 1). Data were averaged from three independent biological experiments with three technical replicates each. Standard errors are shown on the columns. The significance of difference between sets of data was determined by one-way analysis of variance (ANOVA) following Duncan’s multiple range tests; a *p*-value of less than 0.05 was considered significant. Different letters indicate significant differences.

Exogenous application of the NO donor GSNO to the roots during the conversion stage (in parallel with cytokinin) significantly decreased the relative expression of AtPAO5 (already from 24 h cytokinin induction onward) and the shoot regeneration efficiency (Figure 7). Therefore, uncontrolled NO accumulation might act negatively on the regeneration process upstream of AtPAO5.

## 4. Discussion

The regeneration of plants from various explants/tissues via de novo shoot organogenesis in most cases involves callus formation. In addition to plant hormones, the role of polyamines (PAs) has been reported in the process [46,47]. The lateral root primordia (LRPs) of Arabidopsis can be directly converted to shoot meristems (SMs) by the application of exogenous cytokinin, without the need for callus development. In an earlier paper, we reported that PAs are also involved in this meristem conversion pathway [7]. It was found that the level of PAs, particularly spermidine (Spd), increased during the conversion, and the application of exogenous Spd could improve the SM regeneration efficiency. This correlated with the increased metabolism of PAs, as showed by the augmented relative expression of genes involved in PA synthesis as well as catabolism [7]. The expression of the gene coding for the PA catabolic enzyme POLYAMINE OXIDASE 5 (AtPAO5) was observed to be particularly high during this process. This observation agrees with that of Alabdallah and co-workers [19], who reported that AtPAO5 expresses in the root vasculature including the pericycle, where LRP formation starts [48], as well as in the root meristem region. Furthermore, they also demonstrated that cytokinin treatment augments AtPAO5 promoter activity especially in the same root regions [19]. Here, we demonstrate that AtPAO5 expression not only correlates with the cytokinin treatment but is needed for the LRP-to-SM conversion since the loss of AtPAO5 function significantly decreased the success rate of the process (Figure 1). It is also corroborated by our earlier observation that the over-expression of the gene increased the efficiency of LRP-to-SM conversion [7].

How might AtPAO5 be involved into the in vitro shoot regeneration pathway? The by-products of PA catabolism, such as H_2_O_2_, are known to influence various physiological and developmental processes, including in vitro regeneration (for a recent review, [16]). However, the LRP-to-SM conversion was facilitated only by the ectopic expression of AtPAO5 but not that of *AtPAO2*, although both produce H_2_O_2_ [7]. The reason can be that AtPAO5 was shown to differ from the other four Arabidopsis PAO enzymes. It was shown to have a stronger dehydrogenase than oxidase activity having high affinity for T-Spm as a substrate [20,49]. Furthermore, the *pao5-2* knockout mutant has increased sensitivity to T-Spm but not to other polyamines [50]. Thus, this enzyme, in contrast to the other four in Arabidopsis, does not produce excess H_2_O_2_ [49] but efficiently controls the T-Spm level, which is necessary for normal growth [19,20]. In agreement, while T-Spm-treatment of wild-type roots phenocopied the low shoot regeneration capacity of the *pao5-2* mutant, exposing the roots to H_2_O_2_ did not affect the regeneration efficiency (Figure 1 and Figure 2). This is further corroborated by the fact that *pao5-2* and wild-type plants did not display a difference in ROS accumulation during the conversion of LRP to SM (Supplementary Figure S2).

Alterations in T-Spm homeostasis influence a wide range of cellular processes as shown by the gene expression analysis of T-Spm-treated wild-type and *pao5-2* plants [50]. Histological analyses revealed that the AtPAO5-controlled T-Spm level specifically contributes to the differentiation of the vascular system [19,50]. Alabdallah and colleagues [19] proved that AtPAO5 is required to maintain the proper auxin and cytokinin signalling underlying xylem differentiation. Among others, they investigated the expression of A-type ARRs (*ARR4,5,7,15,16*), which are negative feedback regulators of cytokinin signalling, in AtPAO5 overexpressing or *pao5-2* mutant seedlings. It was reported that although there was no difference in the expression of these genes under normal growth conditions, they were less induced in the overexpressor and more in the mutant plantlets in comparison to the wild type when the seedlings were treated by cytokinin. Interestingly, the AtGLB1 and 2 haemoglobins were found to downregulate the same ARR repressors of the cytokinin signalling pathway, and thus increased the cytokinin sensitivity and enhanced the shoot regeneration capacity of the roots [12]. To establish a potential link between AtPAO5 and AtGLBs during the cytokinin-induced SM regeneration process, we investigated the expression of *AtGLB1* and *2*. It was found that both genes, but especially *AtGLB1*, were strongly expressed at the promeristem stages of the LRP-to-SM conversion in the untreated wild-type roots (Figure 3) but neither in the *pao5-2* nor in T-Spm-treated ones. Furthermore, the expression of A-type ARR genes was upregulated in *pao5-2* roots. These indicate that AtPAO5 acts upstream of AtGLBs controlling cytokinin sensitivity, likely via maintaining a permissive T-Spm level.

The signalling pathways on which T-Spm and haemoglobins might regulate gene expression is still unknown. Haemoglobins are known scavengers of NO [21] and NO was shown to mediate cytokinin action on gene regulation [13]. Therefore, one possibility would be that AtGLBs prevent the negative feedback regulation of cytokinin signalling via controlling the NO level. However, exogenous application of NO did not alter the cytokinin-responsive expression of *ARR5* in Arabidopsis, which suggests another way of action for NO in cytokinin responses [35]. The effect of NO on cytokinin signalling can be synergistic or antagonistic [45]. Furthermore, it was shown in several studies that NO metabolism can be affected by polyamines and their catabolism [37,40,41] and polyamine metabolism might also be influenced by NO [51]. In salt-stressed sunflower seedlings, NO augmented the Spm level, upregulating PA synthesis with a parallel lowering of PAO activity [51]. We found that the NO donor GSNO lowered AtPAO5 expression and decreased the efficiency of direct SM formation (Figure 7) indicating that NO may negatively regulate cytokinin sensitivity upstream of AtPAO5. However, NO might indirectly affect the process only at high concentrations and our observation can be less relevant under physiological conditions. It is supported by the fact that the endogenous NO level showed no strong correlation with the regeneration process nor with the AtPAO5 level (Figure 6).

## 5. Conclusions

**Figure 8:**
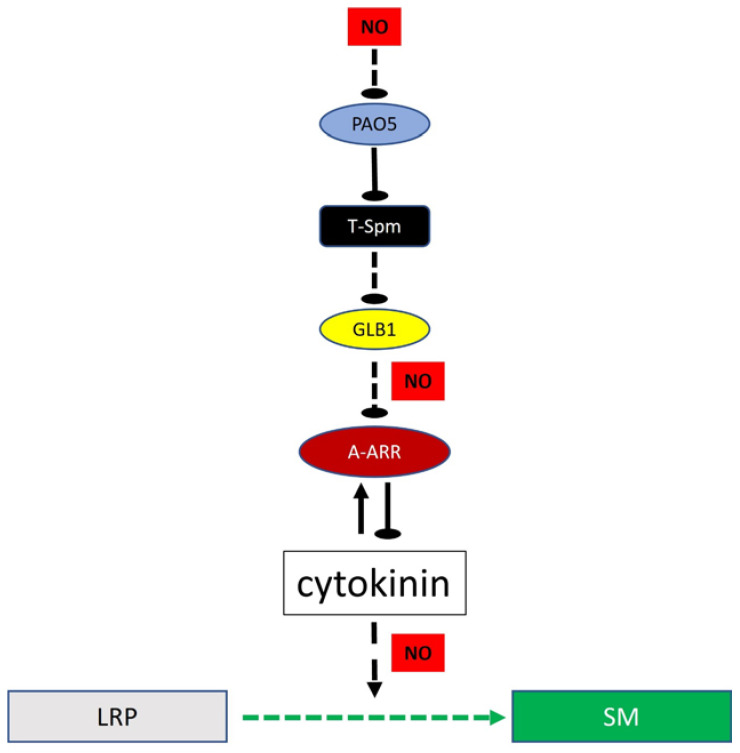
A hypothetical model showing how the Arabidopsis polyamine oxidase 5 (AtPAO5) might influence the cytokinin-induced conversion of lateral root primordia (LRPs) into shoot meristems (SMs). T-Spm—thermospermine; AtGLB1—class 3 haemoglobin; NO—nitric oxide; A-ARR—typeA arabidopsis response regulators. The pointed arrows indicate activation, the round-pointed ones inactivation, the dashed lines indicate indirect interaction.

The AtPAO5-maintained T-Spm homeostasis is needed for efficient cytokinin-induced shoot meristem formation from lateral root primordia. Our experimental data show that high T-Spm level negatively controls, in a yet unknown way, the expression of the *AtGLB1* and *2* class 3 haemoglobin genes. These haemoglobins are known to contribute to cytokinin sensitivity. They indirectly downregulate the expression of the negative feed-back regulators of cytokinin signalling, the A-type ARR genes. It was found that in the absence of AtPAO5, the T-Spm level increased, the expression of the *AtGLB1/2* genes decreased, while that of coding for A-type ARRs augmented and the cytokinin sensitivity was lowered. Although this might depend on the NO-scavenging activity of AtGLBs, we could not provide evidence for that, but rather found that a high NO level inhibited AtPAO5 expression and shoot regeneration. Our findings are summarized in Figure 8. An important task that remains for the future is naming the elements of the signalling steps that link T-Spm and AtGLB1/2 to gene expression regulation. Transcriptomics, promoter analysis, and characterization of further mutants might help to fill in the gaps in the model. These studies can lead to a better understanding and overcoming of the recalcitrance of certain genotypes/explants to cytokinin-induced shoot regeneration.

## Data Availability

Data sharing is not applicable to this article.

## Supplementary Materials

The following supporting information can be downloaded at: https://www.mdpi.com/article/10.3390/plants12030454/s1, Figure S1: Representative images of pixel intensity measurement; Figure S2: ROS level does not differ in the roots of WT (Col) and pao5-2 mutant plants during the formation of shoot meristem from lateral root primordia; Table S1: Sequences of the oligonucleotide primers used in the qPCR; Table S2: WT pao5-2 NO raw data; Table S3: WT pao5-2 ROS raw data.
