# Supplementary material for: Absence of Arabidopsis Polyamine Oxidase 5 Influences the Cytokinin-Induced Shoot Meristem Formation from Lateral Root Primordia
Source: Plants (Basel). 2023 Jan 18;12(3):454. doi: 10.3390/plants12030454 (PMC9920396; doi:10.3390/plants12030454)
Supplement: Supplementary file 1 [file plants-12-00454-s001.zip › Supplementary Table 1.docx]

**Supplementary Table 1.** Sequences of the oligonucleotide primers used in the qPCR

| F: 5' - TCGGTGGAAGCCCTGTTTAT- 3'  R: 5' - CGGCGTCGGATTGAGAGAT- 3' | *AtPAO5 (AT4G29720)* |
| --- | --- |
| F: 5' - ACAAAGGCAAAGGCAACTTC- 3'  R: 5' - CCACATACATCTCGGTTTCGT- 3' | *AtNIA1 (AT1G77760)* |
| F: 5' - GCGTGGTGTCCCTCTCTG- 3'  R: 5' - TGATGCTCGTTCCGTATTTG- 3' | *AtNIA2 (AT1G37130)* |
| F: 5' - CGCAAGTGCTATGCTCGTCTT- 3'  R: 5' - GCCTCAACTGGTTGCTGTGA - 3' | *AtUBQ1 (AT3G52590)* |
| F: 5' - ATTCCGATAGTCGACCAAGC- 3'  R: 5' - AACATCAACATCTGGGTCTTCA - 3' | *AtPP2A3 (AT1G13320.1)* |
| F: 5' - AGCCTCACGCAATGTCTGTT- 3'  R: 5' - TTTCCCTGTTTTCCTCAGTTG- 3' | *AtGLB1 (AT2G16060)* |
| F: 5' - TGAAGTCCCTCACAACAATCC- 3'  R: 5' - TCAGCCACTACCACCTTTCC- 3' | *AtGLB2 (AT3G10520)* |
| F: 5' - ACTGATGGCGGTGTTGACTA- 3'  R: 5' - TTGGAACGGACGAGTTGATA- 3' | *AtGSNOR1 (AT5G43940)* |
| F: 5’- TTCTCATCCTCTCCTCACAGTTAC- 3’  R: 5’- CACCGACGCTCATCATCTCC- 3’ | *AtARR4*  *(AT1G10470)* |
| F: 5’- ATGTCCTGATTCTTTCGGCTTAC- 3’  R: 5’- AACCCATCTTTGTCACTCTTG- 3’ | *AtARR5*  *(AT3G48100)* |
| F: 5’- TATGTCACCTAACCTAACTTC- 3’  R: 5’- ACCGCCATTGTCAAACTCAGA- 3’ | *AtARR7*  *(AT1G19050)* |
| F:5’-ATCTCCATCATCATCATCAAC-3’ R: 5’- ATCTCCATCATCATCATCAAC- 3’ | *AtARR15*  *(AT1G74890)* |
| F:5’-ATCTCTTCCCCATTGCTCTC-3’ R: 5’- TCTCTGGTGATGGTATTTATGATG- 3’ | *AtARR16*  *(AT2G40670)* |
