# Supplementary figures and images for: Absence of Arabidopsis Polyamine Oxidase 5 Influences the Cytokinin-Induced Shoot Meristem Formation from Lateral Root Primordia
Source: Plants (Basel). 2023 Jan 18;12(3):454. doi: 10.3390/plants12030454 (PMC9920396; doi:10.3390/plants12030454)

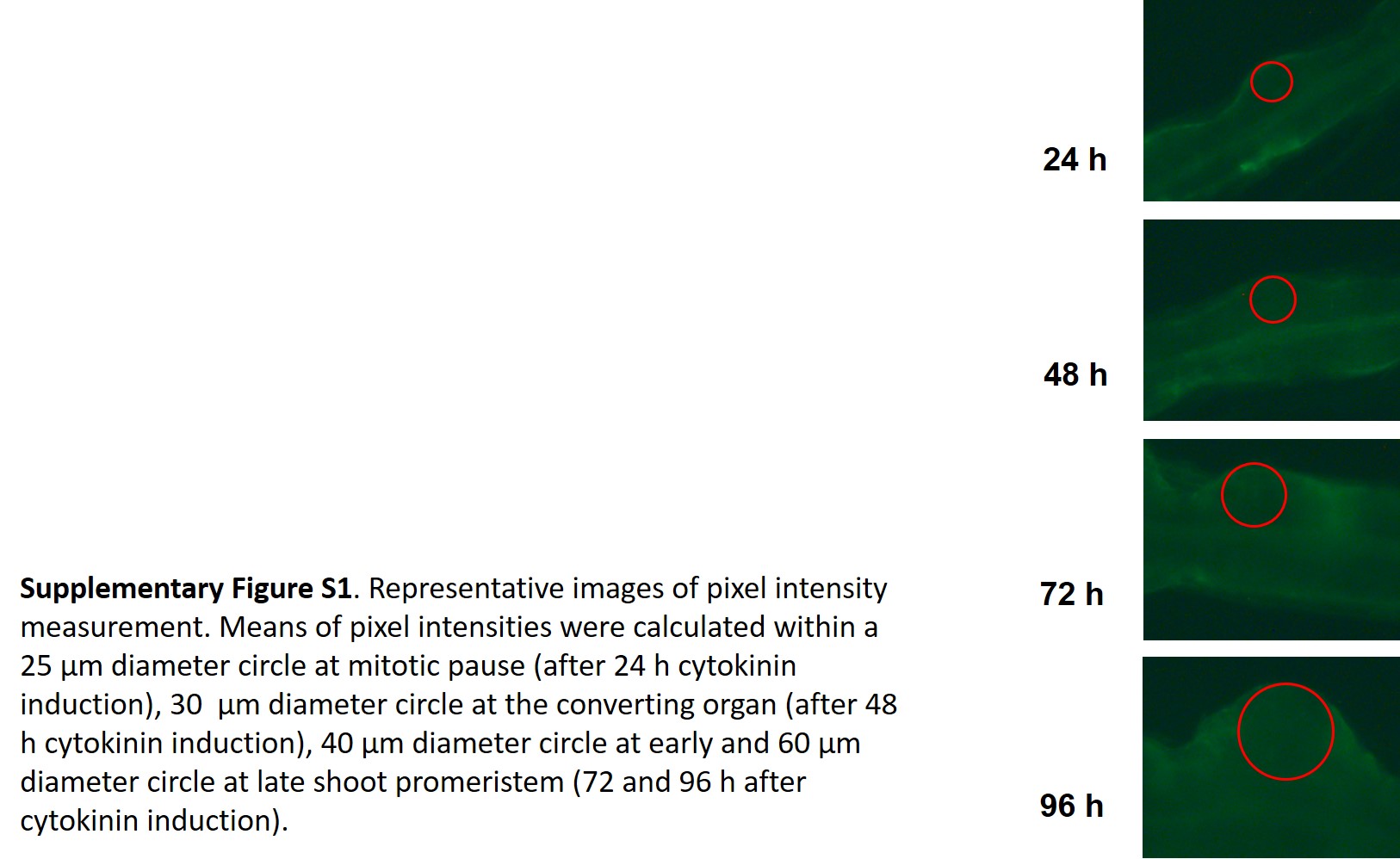

Supplement: Supplementary file 1 [file plants-12-00454-s001.zip › Suppl Fig S1.jpg]

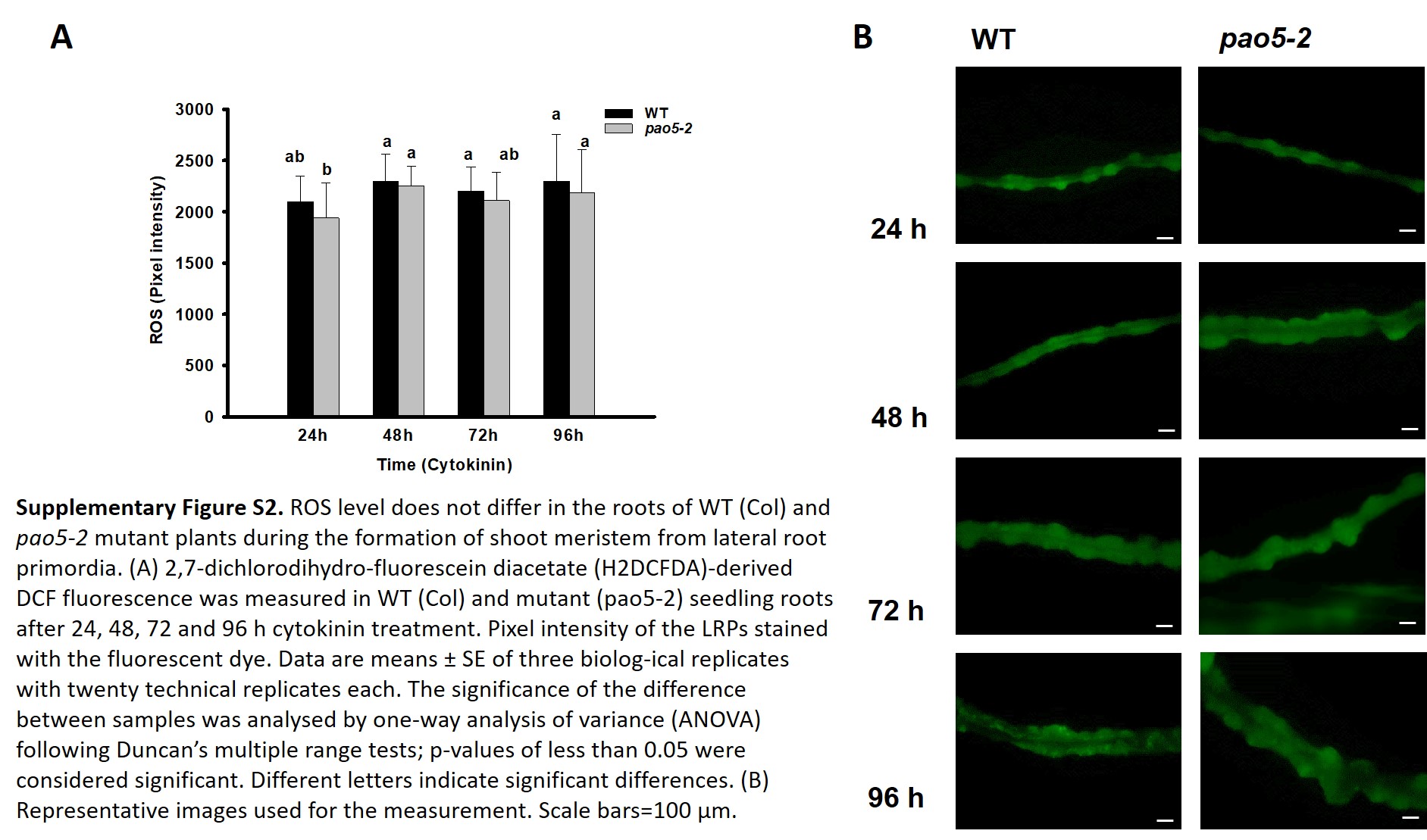

Supplement: Supplementary file 1 [file plants-12-00454-s001.zip › Suppl Fig S2.jpg]
